# First‐Trimester CBC‐Derived Inflammatory Indices for Prediction of Miscarriage: A Retrospective Cohort Study

**DOI:** 10.1002/jcla.70365

**Published:** 2026-09-27

**Authors:** Suchada Wattanaploy, Suchaya Luewan, Theera Tongsong

**Affiliations:** ^1^ Department of Obstetrics and Gynecology, Faculty of Medicine Chiang Mai University Chiang Mai Thailand; ^2^ Fetal Center, Faculty of Medicine Chiang Mai University Chiang Mai Thailand

**Keywords:** CBC‐derived inflammatory indices, first trimester, miscarriage, neutrophil‐to‐lymphocyte ratio, platelet‐to‐lymphocyte ratio, pregnancy, systemic immune‐inflammation index, systemic inflammation response index

## Abstract

**Objective:**

To develop and internally validate a predictive model for miscarriage using clinical factors combined with CBC‐derived inflammatory indices, including the neutrophil‐to‐lymphocyte ratio (NLR), platelet‐to‐lymphocyte ratio (PLR), systemic immune‐inflammation index (SII), and systemic inflammation response index (SIRI).

**Methods:**

This retrospective cohort study included Thai women with singleton pregnancies who attended their first antenatal visit during the first trimester between January 2020 and December 2025. Eligible participants had available CBC results and pregnancy outcomes. Associations between CBC‐derived indices and subsequent miscarriage were evaluated, and predictive models were developed. Model discrimination was assessed using the area under the curve (AUC).

**Results:**

A total of 796 women were included, comprising 118 with subsequent miscarriage and 678 with ongoing pregnancies. Gestational age was significantly lower among the study group (7.32 ± 1.61 vs. 8.69 ± 2.07 weeks, *p* < 0.001). Women who miscarried had significantly higher lymphocyte counts but lower neutrophil counts and NLR than controls. A clinical model based on maternal age and gestational age demonstrated modest discrimination (AUC, 0.741). A penalized model incorporating maternal age, gestational age, NLR, and SII significantly improved predictive performance (AUC, 0.781; *p* = 0.001). At a predicted probability threshold of 0.15, the model achieved a sensitivity of 77.1% and a specificity of 70.0%.

**Conclusion:**

Maternal age and gestational age were significant predictors of miscarriage, and the first‐trimester combination model provided significant improvement in discrimination. Because these indices are routinely derived from standard CBC testing without additional cost, they may potentially serve as practical adjuncts to clinical risk factors for early miscarriage risk stratification after validation.

## Graphical abstract

Maternal age was an independent predictor of miscarriage, and first‐trimester NLR and SII provided significant incremental predictive value. Because these indices are routinely derived from standard CBC testing without additional cost, they may serve as practical adjuncts to clinical risk factors for early miscarriage risk stratification.

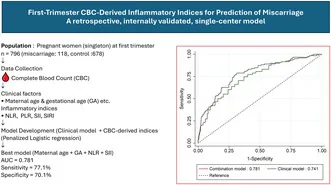

## 1 Introduction

Miscarriage, defined as the spontaneous loss of pregnancy before 20 weeks of gestation, remains the most common complication in early pregnancy, affecting up to 20% of clinically recognized pregnancies. The psychological and physical impact of miscarriage is profound, underscoring the need for early identification of women at increased risk. Such identification may facilitate appropriate counseling, closer clinical surveillance, and timely evaluation of potentially modifiable risk factors or underlying causes in high‐risk women. Despite advances in obstetric care, predicting miscarriage remains challenging due to the multifactorial nature of its etiology, which includes genetic, anatomical, endocrine, immunological, and environmental factors [1, 2, 3].

Recent studies have investigated the predictive value of CBC‐derived inflammatory indices, including the neutrophil‐to‐lymphocyte ratio (NLR), platelet‐to‐lymphocyte ratio (PLR), and monocyte‐to‐lymphocyte ratio (MLR), for miscarriage. These readily available and inexpensive biomarkers reflect systemic inflammation, which may play a role in the pathogenesis of pregnancy loss. Several studies have demonstrated significant associations between altered CBC‐derived inflammatory markers and first‐trimester miscarriage. Lower red blood cell counts and mean platelet volume, together with altered NLR and PLR, have been associated with miscarriage, although their predictive performance has generally been only moderate [4, 5, 6]. Composite CBC‐derived inflammatory indices, particularly the systemic immune‐inflammatory index (SII) and systemic inflammatory response index (SIRI), have also been evaluated for predicting miscarriage. Among these, SIRI has demonstrated superior predictive performance compared with NLR, PLR, and MLR [7].

However, most previous studies have focused on selected populations, such as women undergoing assisted reproductive technology or those with immune disorders, and have not fully explored the predictive utility of routinely available CBC‐derived inflammatory indices [3, 8]. Moreover, limited external validation and clinical implementation have restricted the generalizability of existing models [2]. The inconsistent findings across studies further suggest that inflammatory biomarkers alone are insufficient for accurate risk prediction, highlighting the need for multifactorial models integrating both clinical and laboratory parameters [1, 7, 9, 10, 11]. Therefore, this study was conducted among asymptomatic women before the onset of miscarriage manifestations, with the integration of CBC indices with clinical risk factors to develop a predictive model with internal validation and calibration. The primary objective of this study was to develop and validate predictive models for miscarriage using complete blood count (CBC)‐derived inflammatory indices (MLR, NLR, PLR, SII, and SIRI) in the first trimester combined with clinical risk factors among low‐risk pregnant women (no chronic underlying medical disease) at their initial first‐trimester antenatal visit, and to evaluate the predictive performance of the developed models.

## 2 Patients and Methods

A retrospective cohort study was conducted among pregnant women who received antenatal care and delivered at Maharaj Nakorn Chiang Mai Hospital, Thailand, between January 2020 and December 2025. The study protocol was approved by the Institutional Review Board of the Faculty of Medicine, Chiang Mai University, and was conducted in accordance with the ethical principles of the Declaration of Helsinki and all applicable regulatory requirements (Research ID: 0857; Study Code: OBG‐2568‐0857; approval date: December 11, 2025). After ethical approval, the data were assessed and retrieved from the hospital's electronic medical records and the Maternal‐Fetal Medicine Unit database.

Eligible participants met the following inclusion criteria: (1) women aged > 20 years with singleton pregnancies; (2) complete blood count (CBC) results available during the first trimester (6–14 weeks of gestation); (3) documented normal pregnancy and accurate gestational age at enrollment, defined as an intrauterine pregnancy with fetal heart activity and a crown–rump length appropriate for gestational age, without suspected major fetal anomalies detectable during the first trimester; and (4) documented pregnancy outcomes with respect to miscarriage. The diagnosis of miscarriage was based on the clinical assessment of the treating physician, as documented in the patients’ complete medical records. Women were excluded if they had (1) multifetal pregnancy, (2) unavailable pregnancy outcomes, (3) fetal structural or chromosomal anomalies, when such information was available; (4) a history of threatened abortion or induced abortion in the current pregnancy; (5) chronic medical disease such as inflammatory or autoimmune disorders; or (6) fever or an acute infection at the time of CBC assessment. (7) Conception by assisted reproductive technology (ART), which might potentially influence inflammatory responses.

Socio‐demographic and clinical data extracted from the medical records included maternal age, ethnicity, pre‐pregnancy weight, body mass index (BMI), parity, gestational age, first‐trimester ultrasound findings, smoking status, alcohol consumption, history of previous miscarriage, and chronic medical conditions. Complete blood count (CBC) results obtained at the initial antenatal visit (6–14 weeks' gestation) were retrieved for all participants. The following CBC‐IIs were calculated: The Systemic Immune‐Inflammation Index (SII) = (platelet count × neutrophil count)/lymphocyte count; the neutrophil‐to‐lymphocyte ratio (NLR) = neutrophil count/lymphocyte count; the monocyte‐to‐lymphocyte ratio (MLR) = monocyte count/lymphocyte count; the platelet‐to‐lymphocyte ratio (PLR) = platelet count/lymphocyte count; and the Systemic Inflammation Response Index (SIRI) = (neutrophil count × monocyte count)/lymphocyte count. The CBC tests were performed as part of routine antenatal care in the Clinical Pathology Laboratory of Maharaj Nakorn Chiang Mai Hospital. CBC parameters, including neutrophil, lymphocyte, monocyte, and platelet counts, were measured using an automated hematology analyzer with five‐part differential capability. As this was a retrospective study conducted over six years, the specific analyzer model, reagent lot, and calibration records for each test could not be retrospectively retrieved with certainty. However, the laboratory maintained standardized testing protocols throughout the study period and adhered to routine internal quality control, including daily analysis of multi‐level commercial control materials, together with participation in an external quality assurance/proficiency testing program, consistent with the laboratory's accreditation requirements. No study‐specific deviations from routine clinical testing were introduced. The primary outcome was miscarriage, defined as spontaneous pregnancy loss before 20 completed weeks of gestation. Extracted data were reviewed, cleaned, and validated by the study investigators prior to analysis. Participants were subsequently classified into two groups according to pregnancy outcome: The miscarriage group and the non‐miscarriage (control) group, comprising pregnancies that continued beyond 20 weeks of gestation, regardless of the final pregnancy outcome.

The sample size for prediction model development was determined using the events‐per‐variable approach. Assuming a miscarriage rate of 15%, a minimum of 10 events per predictor parameter was targeted for a multivariable logistic regression model including approximately 10 candidate predictors, comprising CBC‐IIs (e.g., SII, NLR, PLR, and MLR) and clinical risk factors. Accordingly, at least 100 miscarriage events were required to minimize model overfitting and ensure reliable parameter estimation, corresponding to a minimum total sample size of approximately 667 participants.

### 2.1 Statistical Analysis

Statistical analyses were performed using Stata version 16 (StataCorp LLC, College Station, TX, USA). Baseline demographic characteristics and complete blood count‐derived inflammatory indices (CBC‐IIs) were compared between groups using the Student's *t*‐test for continuous variables and the chi‐square test for categorical variables, as appropriate. Univariable and multivariable logistic regression analyses were conducted to estimate crude and adjusted odds ratios (ORs) with corresponding 95% confidence intervals (CIs) for clinical risk factors and CBC‐IIs associated with miscarriage. Predictive models were subsequently developed using binary logistic regression, with clinical risk factors and CBC‐IIs entered as candidate predictors as follows. A multivariable logistic regression model was initially developed using prespecified clinical predictors, including maternal age, BMI, parity, gestational age, and history of miscarriage. The linearity assumption for continuous predictors was assessed by examining their functional relationships with the log odds of miscarriage using restricted cubic spline functions. Nonlinearity was assessed by comparing the spline‐based models with models containing the corresponding predictors as linear terms. To achieve a parsimonious clinical model, variables with limited contribution to model performance were excluded, resulting in a model containing maternal age and gestational age. CBC‐derived inflammatory indices, including NLR, PLR, MLR, SII, and SIRI, were subsequently added to the clinical predictors. Because of potential collinearity among the CBC‐derived inflammatory indices, penalized logistic regression using an elastic‐net approach was applied to identify the most informative predictors and reduce model complexity. Model discrimination was assessed by the area under the receiver operating characteristic (ROC) curve (AUC), and differences in AUCs between models were compared using the DeLong test. The final model was internally validated using bootstrap resampling to assess optimism‐corrected model performance and calibration. Diagnostic performance measures, including sensitivity, specificity, positive predictive value, negative predictive value, positive likelihood ratio, and negative likelihood ratio, were calculated at the optimal probability threshold, based on Youden's index. All statistical tests were two‐sided, and a *p*‐value < 0.05 was considered statistically significant.

## 3 Results

A total of 796 pregnancies were included, comprising 678 controls and 118 cases of miscarriage as presented in Figure 1. Note that approximately 30% (338 cases) were excluded because of incomplete clinical data, with most of these cases having ended their pregnancies elsewhere. Women in the miscarriage group were significantly older than those with normal pregnancies (35.49 ± 5.22 vs. 31.68 ± 4.68 years, *p* < 0.001). In addition, gestational age at the first antenatal care visit was significantly lower among pregnancies that resulted in miscarriage (7.32 ± 1.61 vs. 8.69 ± 2.07 weeks, *p* < 0.001), as presented in Table 1. No significant differences were observed between groups with respect to pre‐pregnancy BMI (24.07 ± 5.53 vs. 24.05 ± 5.40 kg/m^2^, *p* = 0.985), parity status (*p* = 0.318), smoking (*p* = 0.600), alcohol use (*p* = 1.000), or history of miscarriage in previous pregnancy (*p* = 0.156). Overall, pregnancies resulting in miscarriage were characterized by advanced maternal age and earlier gestational age at the first antenatal visit, whereas other baseline maternal and obstetric characteristics were comparable between groups.

**FIGURE 1:**
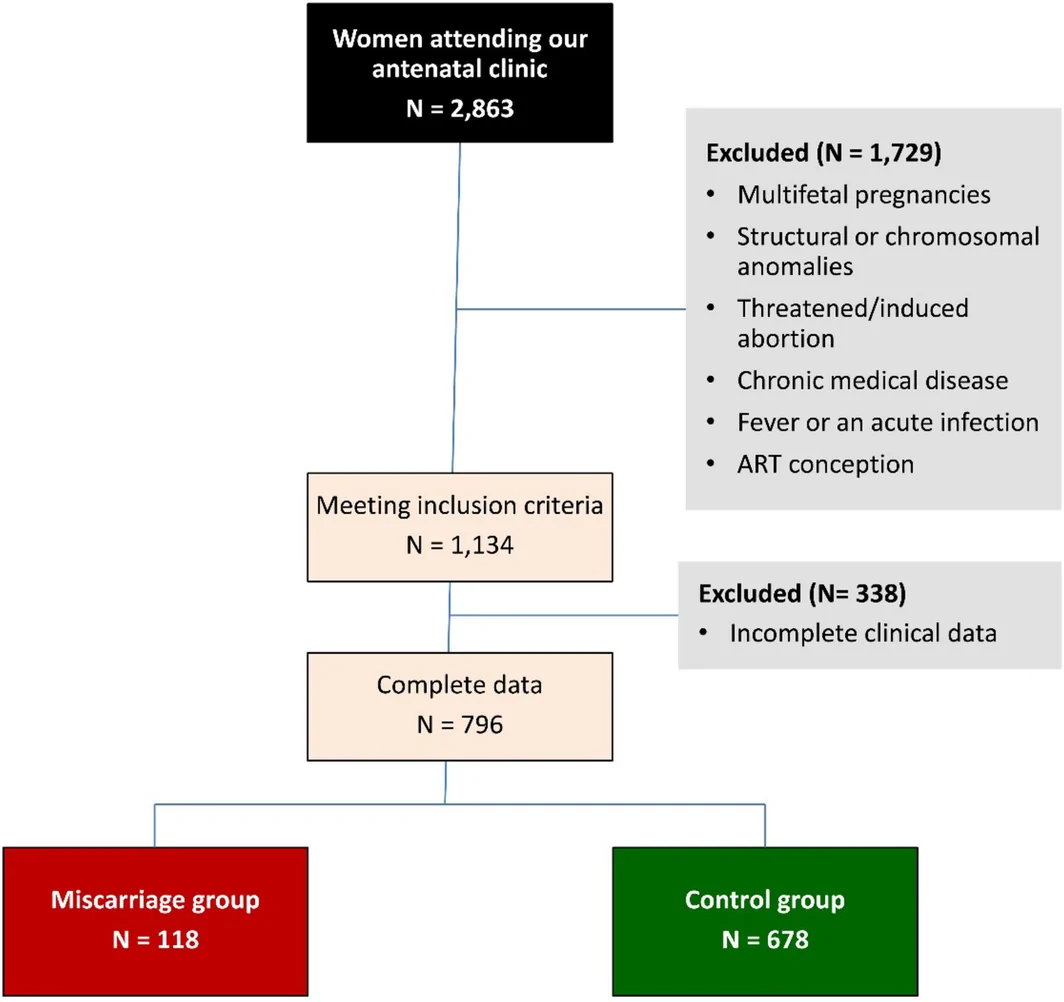
Flowchart of patient recruitment.

**TABLE 1:** Baseline characteristics of the study.

Characteristics	Control (*n* = 678)	Case (*n* = 118)	*p* *
Maternal characteristics			
Age (years)	31.68 ± 4.68	35.49 ± 5.22	< 0.001
BMI (kg/m^2^)	24.05 ± 5.40	24.07 ± 5.53	0.985
Gestational age at first ANC (weeks)	8.69 ± 2.07	7.32 ± 1.61	< 0.001
Obstetric history			
Nulliparous	384 (56.6%)	61 (51.7%)	0.318
Smoking	6 (0.9%)	0 (0.0%)	0.600
Alcohol use	1 (0.1%)	0 (0.0%)	1.000
History of prior miscarriage	81 (11.9%)	18 (15.3%)	0.315

Abbreviations: BMI, body mass index; CBC, complete blood count; GA, gestational age; SD, standard deviation.

* Student's *t*‐test or Chi‐square test.

Compared with normal pregnancies, pregnancies resulting in miscarriage had significantly lower neutrophil counts (6292 ± 1735 vs. 7207 ± 1948/μL, *p* < 0.001) and higher lymphocyte counts (2358 ± 587 vs. 2236 ± 593/μL, *p* = 0.039). No significant differences were observed in hemoglobin, hematocrit, mean corpuscular volume, platelet count, or monocyte count between the two groups, as presented in Table 2. Among the inflammatory indices, the miscarriage group exhibited significantly lower NLR, SII, and SIRI compared with normal pregnancies. In contrast, PLR and MLR did not differ significantly between groups (*p* = 0.557 and *p* = 0.184, respectively). Overall, pregnancies with miscarriage were characterized by lower neutrophil‐related inflammatory markers, particularly NLR, SII, and SIRI, suggesting an association between altered systemic inflammatory status in early pregnancy and miscarriage risk.

**TABLE 2:** Comparisons of hematologic parameters between the two groups.

Characteristics	Control (*n* = 678) Median (IQR)	Case (*n* = 118) Median (IQR)	*p* *
Complete blood count			
Hemoglobin (g/dL)	12.5 (11.6–13.0)	12.5 (11.7–13.2)	0.215
Hematocrit (%)	37.9 (35.8–39.7)	38.2 (36.0–40.0)	0.585
MCV (fL)	84.45 (78.5–88.5)	84.40 (76.00–88.30)	0.596
Platelet count (/μL)	285,500 (245,000–324,000)	288,500 (253,000–336,000)	0.162
Neutrophil count (/μL)	7037 (5742–8417)	6083 (4963–7070)	< 0.001
Lymphocyte count (/μL)	2176 (1828–2558)	2350 (1930–2732)	0.019
Monocyte count (/μL)	462 (372–571)	464 (388–574)	0.732
Inflammatory indices			
NLR	3.20 (2.58–3.97)	2.55 (2.09–3.32)	< 0.001
PLR	128.28 (108.94–154.73)	127.10 (105.98–154.68)	0.664
MLR	0.21 (0.17–0.26)	0.20 (0.17–0.24)	0.099
SII	900.45 (696.07–1170.16)	825.10 (585.42–1086.81)	0.007
SIRI	1482.10 (1071.01–2036.60)	1324.74 (914.03–1696.23)	0.001

Abbreviations: SII, Systemic Immune‐Inflammation Index; SIRI, Systemic Inflammation Response Index; NLR, Neutrophil‐to‐Lymphocyte Ratio; PLR, Platelet‐to‐Lymphocyte Ratio.

* Mann–Whitney U test.

On univariate analysis, among the clinical risk factors evaluated, maternal age was the only significant predictor of miscarriage, with an OR of 1.18 (95% CI 1.13–1.23). Maternal age also demonstrated the best discriminatory performance with an AUC of 0.707 (95% CI 0.653–0.761). In contrast, pre‐pregnancy BMI, parity, smoking status, and previous history of miscarriage were not significantly associated with the outcome (Table 3). Among laboratory markers, NLR showed the strongest predictive ability with an OR of 0.59 (95% CI 0.47–0.73) and provided the highest discriminatory ability with AUC of 0.664 (95% CI 0.608–0.720). Both SII and SIRI were also significantly associated with miscarriage, although their discriminative performance was modest (AUC 0.577 and 0.593, respectively). Hemoglobin, PLR, and MLR were not significantly associated with miscarriage. Overall, maternal age and inflammatory biomarkers, particularly NLR, were the most informative predictors, supporting their inclusion in subsequent multivariable prediction models.

**TABLE 3:** Univariate analysis of potential risk factors in predicting subsequent miscarriage.

	Odds ratio (95% CI)	*p*	AUC of ROC (95% CI)
Clinical risk factors			
Maternal age (year)	1.18 (1.13–1.23)	< 0.001	0.707 (0.653–0.761)
Gestational age (year)	0.65 (0.57–0.74)	< 0.001	0.695 (0.647–0.744)
Pre‐pregnancy BMI	1.00 (0.98–1.02)	0.985	0.519 (0.465–0.574)
Parity (ref. = 0)	1.22 (0.82–1.81)	0.319	0.525 (0.476–0.574)
History of prior miscarriage	1.33 (0.76–2.31)	0.316	0.516 (0.481–0.551)
Hematological factors			
Hemoglobin	1.13 (0.95–1.35)	0.156	0.536 (0.476–0.596)
NLR	0.59 (0.47–0.73)	< 0.001	0.664 (0.608–0.720)
PLR	0.999 (0.994–1.003)	0.557	0.513 (0.454–0.571)
MLR	0.15 (0.01–2.50)	0.184	0.547 (0.491–0.604)
SII	0.9993 (0.9988–0.9999)	0.019	0.577 (0.519–0.635)
SIRI	0.9996 (0.9993–0.9999)	0.014	0.593 (0.537–0.649)

Abbreviations: BMI, body mass index; MLR, Monocyte‐to‐Lymphocyte Ratio; NLR, Neutrophil‐to‐Lymphocyte Ratio; PLR, Platelet‐to‐Lymphocyte Ratio; SD, standard deviation; SII, Systemic Immune‐Inflammation Index; SIRI, Systemic Inflammation Response Index.

Two prediction models were developed and evaluated based on clinical data as presented in Table 4. The full clinical model, which included age, BMI, and history of miscarriage, demonstrated moderate discriminative ability with an AUC of 0.711. Among the clinical variables, age was the only significant predictor (*p* < 0.001), indicating that increasing age was associated with higher odds of miscarriage. BMI (*p* = 0.803) and history of miscarriage (*p* = 0.396) were not significantly associated with the outcome.

**TABLE 4:** Logistic Regression Models for Predicting Miscarriage Using Clinical Predictors.

Miscarriage	Coefficient	Standard Error	z‐statistic	*p*	95% CI
Full clinical model					
Age (year)	0.1804	0.0247	7.31	0.000	0.1321 to 0.2288
BMI (Kg/m^2^)	0.0215	0.0215	1.00	0.318	−0.0207 to 0.0637
Parity	−0.2453	0.2231	−1.10	0.271	−0.6825 to 0.1919
Gestational age (week)	−0.2601	0.0618	−4.21	0.000	−0.3813 to −0.1390
History of prior miscarriage	0.0179	0.3022	0.06	0.953	−0.5744 to 0.6103
Constant	−6.0868	1.0861	−5.60	0.000	−8.2156 to −3.9581
Area under ROC curve (95% CI)	0.7453 (0.6961–0.7944)				
Parsimonious clinical model					
Age (year)	0.1718	0.0233	7.38	0.000	0.1262 to 0.2175
Gestational age (week)	−0.2643	0.0617	−4.29	0.000	−0.3851 to −0.1434
Constant	−5.3507	0.9020	−5.93	0.000	−7.1185 to −3.5829
Area under ROC curve (95% CI)	0.7416 (0.6925 to 0.7906)				

Abbreviations: BMI, body mass index; CI, confidence interval.

A parsimonious clinical model retaining only age showed similar predictive performance (AUC = 0.707). Age remained a significant predictor (*p* < 0.001), suggesting that removal of the non‐significant variables resulted in a more simplified model without substantial loss of discrimination.

The full clinical plus laboratory model, incorporating age, NLR, PLR, MLR, SII, and SIRI, achieved the highest discrimination among the full models, with an AUC of 0.763. In this model, age (*p* < 0.001) and NLR (*p* < 0.001) were significant predictors, whereas PLR, MLR, SII, and SIRI were not statistically significant.

The parsimonious clinical plus laboratory model, consisting of age, NLR, and SII, demonstrated an AUC of 0.761 (95% CI 0.711–0.811), which was comparable to that of the full laboratory model. All retained predictors were statistically significant, including age, NLR, and SII.

Overall, the addition of laboratory inflammatory markers improved model discrimination compared with clinical predictors alone (AUC approximately 0.76 vs. 0.71). The parsimonious clinical‐plus‐laboratory model achieved discrimination similar to the corresponding full model while requiring fewer predictors, supporting its potential utility as a more practical prediction tool.

### 3.1 Predictive Model Based on Clinical and CBC‐Derived Inflammatory Indices

The five CBC‐derived indices demonstrated moderate‐to‐high correlations with one another (Table 5). Given the substantial intercorrelation among these indices, elastic‐net penalized logistic regression was employed to develop the predictive model incorporating clinical risk factors and CBC‐derived indices.

**TABLE 5:** Correlation among CBC‐derived inflammatory indices.

	NLR	PLR	MLR	SII	SIRI
NLR	1.0000				
PLR	0.5843	1.0000			
MLR	0.4983	0.3670	1.0000		
SII	0.8556	0.7231	0.3951	1.0000	
SIRI	0.6993	0.2641	0.8194	0.6597	1.0000

*Note:* All pairwise correlations were statistically significant (p < 0.001).

The penalized logistic regression using an elastic‐net framework with cross‐validation was performed to address potential multicollinearity among candidate predictors. Cross‐validation selected α = 1.0 (lasso penalty) and λ = 0.0008035, resulting in retention of age, gestational age, NLR, and SII, while PLR, MLR, and SIRI were shrunk to zero, providing the final predictive models as presented in Table 6, which demonstrated an apparent AUC of 0.7814 (95% CI 0.7342–0.8282).

**TABLE 6:** Final predictive models for miscarriage based on clinical and CBC predictors.

Predictor	Penalized coefficient (β)	Penalized Odd ratios
Age	0.164177	1.178
GA	−0.222735	0.800
NLR	−0.897949	0.407
SII	0.001472	1.001
Constant	−4.004164	0.018

Receiver operating characteristic (ROC) curve analysis demonstrated significantly improved predictive performance following the addition of CBC markers to clinical predictors, as presented in Figure 2. (The AUC of 0.7814 vs. 0.7416, respectively; DeLong test, *p*‐value = 0.001). The findings suggest that inflammatory indices provide incremental predictive value beyond clinical risk factors alone.

**FIGURE 2:**
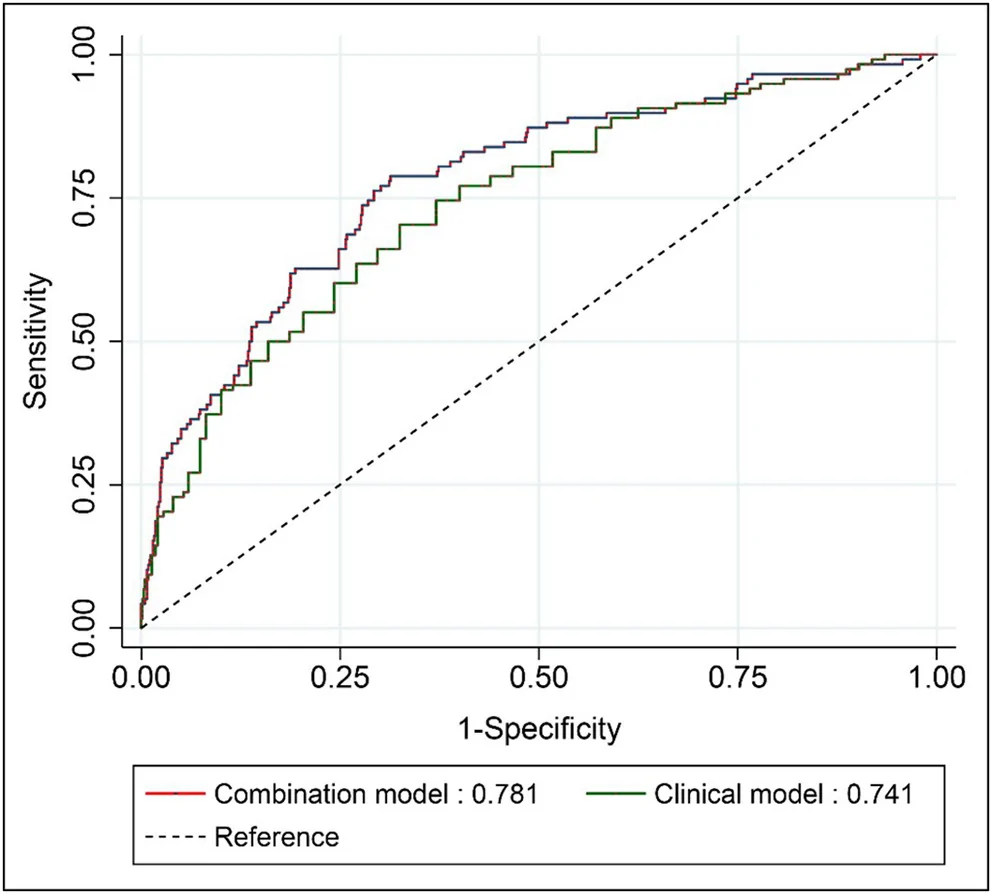
Apparent performance of the predictive model of clinical risk and combined risk in predicting subsequent miscarriage indicates that the combined model demonstrated better discrimination than the clinical model, with an AUC of 0.781 versus 0.741 (DeLong test, *p*‐value 0.001).

The calibration assessment demonstrated good agreement between predicted and observed miscarriage risk, as presented in Figure 3. The model showed satisfactory overall bootstrap performance (optimism‐adjusted) as follows:

- 500 bootstrap resamples
- Mean Brier score = 0.104 (SD = 0.211)
- Bootstrap‐corrected AUC = 0.7808 (95% CI 0.7321–0.8295) (optimism 0.0006)
- E:O ratio = 1.004 (95% CI 0.845–1.162)
- Calibration slope = 1.0222 (95% CI 0.9724–1.0721)
- Calibration in the large = 0.013

**FIGURE 3:**
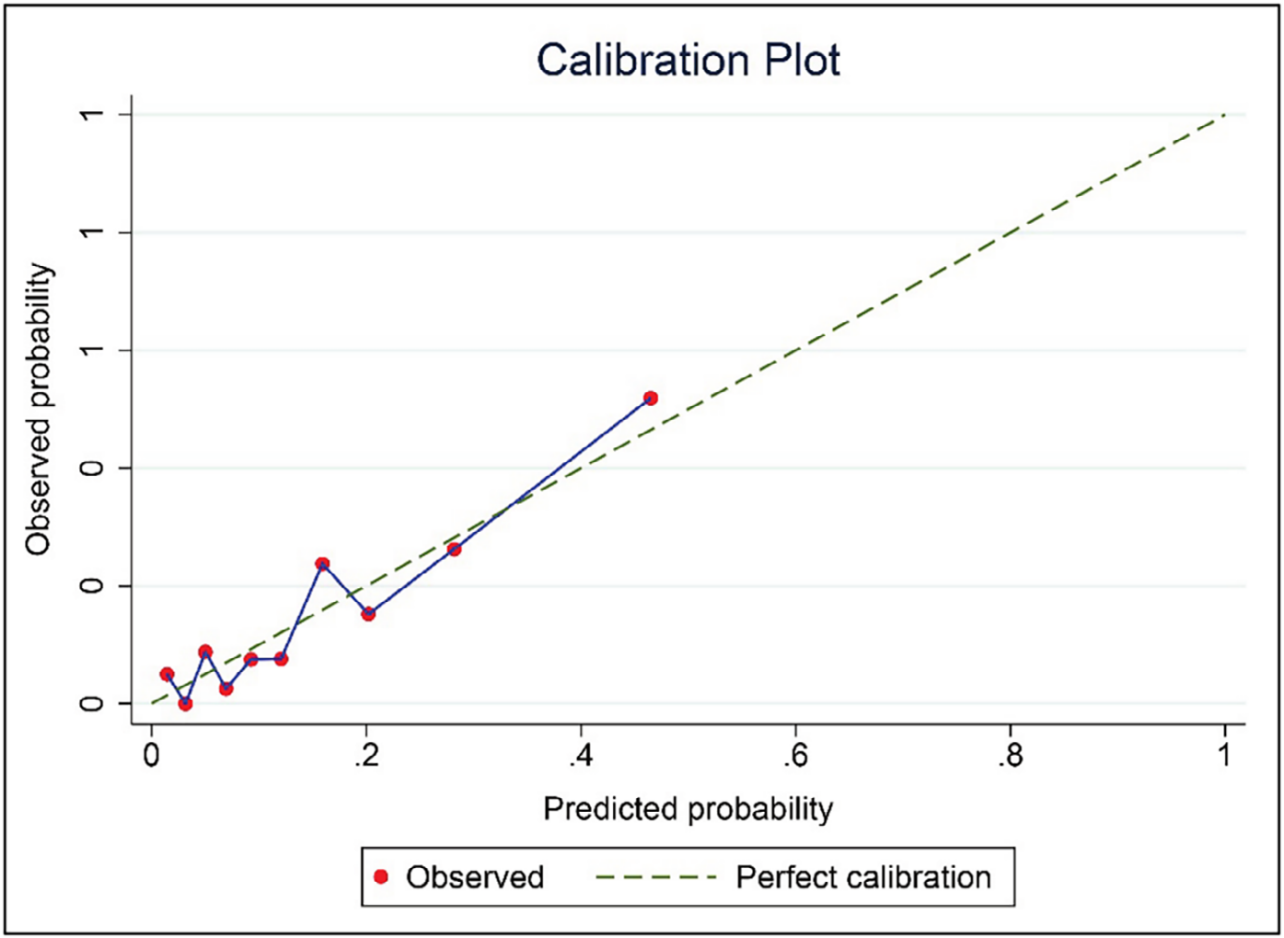
Calibration plot of the final predictive model of the combined risk in predicting subsequent miscarriage (dashed line representing the reference ideal line).

The classification performance of the combined prediction model demonstrated a sensitivity of 71.1% (95% CI 68.5%–84.3%) and a specificity of 70.0% (95% CI 66.3%–73.3%), using a probability cut‐off of 0.15 based on Youden's index, as presented in Table 7. The positive predictive value was 30.8% (95% CI 25.6%–36.5%), while the negative predictive value was 94.6% (95% CI 92.2%–96.4%).

**TABLE 7:** Classification performance of the combined risk model (using cut‐off probability at 0.15).

Group	Predict positive	Predict negative	Total
Miscarriage	91	27	118
Non‐miscarriage	204	473	677
Total	295	500	795

Prognostic indices	Value	95% CI (lower)	95% CI (upper)
Prevalence	14.8%	12.4%	17.5%
Sensitivity	77.1%	68.5%	84.3%
Specificity	70.0%	66.3%	73.3%
Likelihood ratio (+)	2.56	2.20	2.98
Likelihood ratio (−)	0.33	0.23	0.46
Positive predictive value	30.8%	25.6%	36.5%
Negative predictive value	94.6%	92.2%	96.4%

## 4 Discussion

New insights derived from this study are summarized as follows: (1) pregnancies that subsequently resulted in miscarriage exhibited higher first‐trimester lymphocyte counts but lower neutrophil counts than normal pregnancies, leading to a lower NLR value; (2) NLR and SII were identified as significant risk factors for subsequent miscarriage; (3) advanced maternal age was also a significant risk factor for miscarriage and demonstrated moderate predictive performance, with an AUC of 0.707; (4) CBC‐derived inflammatory indices provided significant incremental predictive value beyond maternal age alone in the prediction of miscarriage; and (5) a parsimonious prediction model incorporating maternal age, NLR, and SII achieved an AUC of 0.762, significantly improving the discriminatory performance.

Unlike most previous studies, which did not perform multivariable analyses, the present study adjusted for potential confounding factors, thereby providing a more robust assessment of the significant association between CBC‐derived inflammatory indices and miscarriage risk. It is noteworthy that many published studies have reported only limited discriminatory performance of inflammatory indices when used as standalone predictors [4, 5, 6, 7, 12]. In contrast, our findings suggest that although these indices alone may have modest predictive value, they can significantly enhance the predictive performance of established clinical risk factors when incorporated into a multivariable prediction model.

### 4.1 Plausible Mechanism

We hypothesize that elevated lymphocyte levels in subsequent miscarriage may reflect impaired maternal‐fetal immune tolerance. Successful pregnancy is characterized by an anti‐inflammatory, Th2/Treg‐dominant profile that promotes fetal tolerance [13, 14], whereas miscarriage is associated with Th1 activation, an increased Th1/Th2 ratio, reduced Treg number and function, and enhanced cytotoxic lymphocyte and inflammatory cytokine activity, contributing to trophoblast injury and impaired placental development [15, 16, 17, 18, 19].

Interpretation of the association between CBC‐derived inflammatory markers and miscarriage across published studies should be undertaken with caution, particularly with respect to the timing of blood sampling. When evaluating the predictive performance of inflammatory markers, blood samples should be obtained before the onset of any clinical manifestations of miscarriage, such as vaginal bleeding or uterine contractions. Unlike many previous studies that investigated inflammatory markers in women with threatened miscarriage or recurrent pregnancy loss, the present study collected first‐trimester CBC data before the initiation of the miscarriage process. In several previous studies, CBC measurements were obtained during the course of miscarriage, when inflammatory responses had likely already been activated, resulting in elevated inflammatory indices. This difference in the timing of blood sampling may account for the discrepancy of the findings between our study and many previous reports. Based on our findings, we speculate that there is an “optimal window” of maternal innate immune activation in early pregnancy, characterized by appropriately elevated neutrophil counts and balanced neutrophil‐to‐lymphocyte–derived indices, which is necessary to support implantation, trophoblast invasion, and early placentation. In this framework, pregnancies with relatively reduced neutrophil counts and lower composite indices such as NLR, SII, and SIRI, despite being clinically asymptomatic, reflect an inadequately activated innate immune milieu that fails to provide sufficient support for decidual remodeling and placental development, thereby increasing the risk of early pregnancy loss.

### 4.2 Strengths and Limitations

The strength of this study is that the predictive model incorporated only clinical and laboratory variables that are routinely available at the initial antenatal care visit, without requiring additional investigations or costs. This approach may potentially enhance the practicality and clinical applicability of the model by facilitating early risk stratification for miscarriage using routinely collected data. This study also has several limitations. First, because of its retrospective, single‐center design and the long study period, the analysis relied on data collected during routine clinical practice. Consequently, important variables that are not routinely assessed as part of standard antenatal care were unavailable for analysis or adjustment. For example, information on ultrasound, exact causes of previous abortion, and fetal chromosomal abnormalities, which are well‐established causes of miscarriage and potential confounding factors, could not be evaluated. Second, although the sample size was sufficient to address the primary objective, it was relatively small for evaluating uncommon risk factors, such as cigarette smoking and alcohol consumption. The limited number of participants with these exposures may have reduced the statistical power to detect significant associations in the multivariable analysis. Finally, external validation was not performed.

### 4.3 Future Directions

Further research is warranted to develop practical predictive models with improved discriminatory performance for the early identification of miscarriage risk. Large‐scale prospective studies should be undertaken to evaluate the incremental value of incorporating additional predictors, such as first‐trimester ultrasound features and other well‐established clinical risk factors, into prediction models. Such efforts may contribute to the development of more robust and clinically applicable tools for risk stratification in early pregnancy.

## 5 Conclusion

In this retrospective, single‐center cohort, maternal age and selected first‐trimester CBC‐derived indices, adjusted for gestational age at blood sampling, were associated with subsequent miscarriage. A model incorporating maternal age, gestational age, and CBC‐derived markers demonstrated greater apparent discrimination than a model based on clinical factors alone. However, substantial correlation among the derived indices and the absence of external validation limit the interpretation and generalizability of these findings. Further model development incorporating established ultrasound predictors, followed by independent external validation, is required before any consideration of clinical application.

## Author Contributions

S.W.: Conceptualization, proposal development, methodology, acquisition of data, data validation, manuscript writing; S.L.: Conceptualization, proposal development, methodology, acquisition of data, validation, manuscript editing; T.T.: Data validation, formal analysis and manuscript editing; all authors have read and agreed to the published version of the manuscript.

## Funding

The authors have nothing to report.

## Ethics Statement

The study was conducted in accordance with the Declaration of Helsinki and approved by Research Ethics Committee 5, Faculty of Medicine, Chiang Mai University (Research ID: 0857; Study Code: OBG‐2568‐0857; approval date: December 11, 2025).

## Conflicts of Interest

The authors declare no conflicts of interest.

## Data Availability

The data that support the findings of this study are available from the corresponding author upon reasonable request.
